# A Double-Blind, Placebo-Controlled Study of Adjunctive Topiramate in Adolescents With Co-Occurring Bipolar and Cannabis Use Disorders

**DOI:** 10.1016/j.jaacop.2024.08.002

**Published:** 2024-09-07

**Authors:** Jenni E. Farrow, Melissa P. DelBello, Luis R. Patino, Thomas J. Blom, Jeffrey A. Welge

**Affiliations:** University of Cincinnati College of Medicine, Cincinnati, Ohio

**Keywords:** cannabis, topiramate, bipolar disorder, cannabis use

## Abstract

**Objective:**

To evaluate the efficacy of adjunctive topiramate (TPM) for the treatment of cannabis use disorder in adolescents with bipolar I disorder.

**Method:**

We conducted a 16-week, double-blind, randomized, placebo-controlled investigation of quetiapine plus TPM (median dose = 208 mg) vs quetiapine plus placebo in adolescents with bipolar I and cannabis use disorder. All subjects participated in a Motivational Interview and Compliance Enhancement Therapy. The primary outcome measure was change in weekly cannabis use over a 16-week treatment period using the Timeline Followback. The secondary outcome measure was the baseline-to-endpoint total score change in the Young Mania Rating Scale (YMRS).

**Results:**

A total of 75 participants completed at least 1 post-baseline assessment (TPM = 38, placebo = 37). There was a significant time-by-treatment effect over the 16-week treatment period, with baseline-adjusted treatment differences in favor of the TPM group over time (*p* < .001). Although there was no difference in baseline-to-endpoint YMRS total score change between groups (*p* = .342), there was as significant decline in YMRS total score within both groups (*p* < .0001). There was a significant positive effect for alcohol use (*p* < .001) and nicotine use (*p* = .033) in the TPM group. More participants in the TPM group experienced appetite decrease (*p* = .032) and excitement (*p* = .025). Participants in the placebo group experienced greater weight gain (*p* = .010).

**Conclusion:**

Treatment with TPM adjunctive to quetiapine and a Motivational Interview and Compliance Enhancement Therapy is associated with a greater decrease in cannabis use and less weight gain. TPM is a well-tolerated and efficacious treatment for cannabis use disorder in adolescents with bipolar I disorder.

**Clinical trial registration information:**

Efficacy Study of Quetiapine Plus Topiramate for Reducing Cannabis Consumption and Bipolar Mania; https://clinicaltrials.gov/; NCT00393978.

**Diversity & Inclusion Statement:**

We worked to ensure sex and gender balance in the recruitment of human participants. We worked to ensure race, ethnic, and/or other types of diversity in the recruitment of human participants. We worked to ensure that the study questionnaires were prepared in an inclusive way. One or more of the authors of this paper self-identifies as a member of one or more historically underrepresented racial and/or ethnic groups in science.

## Plain language summary

Cannabis is the most commonly misused drug in adolescents with bipolar disorder, and there is limited prior research supporting the addition of topiramate to motivational enhancement for the treatment of cannabis use disorder. In this study, 75 adolescents and young adults, aged 12-21, with bipolar I disorder participated in a 16-week, double-blind, randomized, placebo-controlled trial of quetiapine plus topiramate versus quetiapine plus placebo treatment for cannabis use disorder. All subjects participated in a Motivational Interview and Compliance Enhancement Therapy. The group receiving topiramate had a greater decrease in the use of cannabis, alcohol, and nicotine, as well as less weight gain. These results suggest that adjunctive topiramate to quetiapine and motivational interview may be helpful in the treatment of cannabis use disorder in adolescents and young adults with bipolar 1 disorder.

Bipolar disorders are severe, chronic, and disabling mental disorders. They share a complex reciprocal relationship with substance use disorders, and the co-occurrence is associated with poorer outcomes. Bipolar disorders are associated with the highest rate of comorbid substance use disorders as compared to any other major mental health disorder, and up to two-thirds of patients with bipolar disorder have a lifetime substance use disorder.^1^ Moreover, adolescents with bipolar disorder have significantly higher lifetime rates of substance use disorders compared with adolescents without bipolar disorder (ie, 34% vs 4%, respectively),^2^ and 48% of hospitalized adolescents with bipolar I disorder either have or will develop a substance use disorder.^3^ Conversely, bipolar disorder is one of the most common non–substance-related mental health disorders in patients with substance use disorders.^4^

In addition, in bipolar disorder, comorbid substance use disorders are associated with worse outcomes. Adults with co-occurring bipolar and substance use disorders experience delayed recovery from mood episodes, increased suicidality, decreased medication adherence, functional impairment, and decreased quality of life.^5^ Correspondingly, adolescents with comorbid bipolar and substance use disorders have a greater lifetime prevalence of suicide attempt and 12-month prevalence of pregnancy, more misconduct requiring police intervention,^6^ and more frequent affective episodes with shorter time to recurrence, and are less likely to be medication adherent^7^ compared with bipolar adolescents without substance use disorders.

In adolescents with bipolar disorder, cannabis is the most common misused drug.^3^ Cannabis use in adults with bipolar disorder is associated with elevated mood and lower global functioning, poor treatment compliance, increased illness severity, a risk of recurrent mood episodes, and, compared with adults with bipolar disorder who do not misuse cannabis, a 1.4-fold greater rate of previous suicide attempts.^8^ Despite this, to our knowledge, there have been no controlled studies specifically evaluating the treatment of adolescents with co-occurring bipolar and cannabis use disorders. In fact, most treatment studies of youth with bipolar disorder exclude those with cannabis use disorders.

Although comorbid bipolar and substance use disorders impose considerable illness burden, there are few studies evaluating treatment for this comorbidity in adolescents. In the only double-blind, placebo-controlled study evaluating adolescents with co-occurring bipolar and substance use disorders, lithium was efficacious for both disorders.^9^ In older adolescents and adults with comorbid bipolar and cannabis use disorders treated with combination lithium and divalproex, 53% either no longer met criteria for active cannabis abuse or achieved full remission.^10^ Finally, in a small non-controlled trial of adjunctive family-focused therapy for adolescents with comorbid bipolar and substance use disorders (most of whom had cannabis use disorders), there were significant improvements in depressive and manic symptoms and global functioning without significant improvement in substance use outcomes.^11^

Topiramate (TPM) is approved by the US Food and Drug Administration (FDA) as an anticonvulsant and for migraine prophylaxis. It facilitates γ-aminobutyric acid (GABA) transmission by binding to GABA-A receptors and inhibits glutamatergic transmission at αamino-3-hydroxy-5-methylisoxazole-4-propionic acid and kainite glutamate receptors, which mediate voltage-dependent sodium and calcium channels. These actions are theorized to confer neurostabilization and to reduce dopamine release in the corticomesolimbic system, which mediates mechanisms involved with reinforcement and reward. In addition, cannabinoid type I receptors, the primary target of cannabis, are highly present on GABAergic interneurons and glutamatergic neurons. Indeed, in adults, TPM is efficacious in treating opiate withdrawal, achieving and maintaining cocaine abstinence, reducing craving and promoting abstinence associated with alcohol, reducing methamphetamine use, maintaining methamphetamine abstinence, and reducing opioid use and craving.^12^

A double-blind, placebo-controlled trial evaluating the efficacy of TPM in children and adolescents suggested that it may be effective for mania, but the results ultimately were inconclusive, as the study was discontinued with only a quarter of the proposed sample enrolled after adult mania trials failed to show efficacy.^13^ To our knowledge, there is only one study examining TPM plus motivational enhancement vs motivational enhancement alone for treating cannabis use among adolescents. In this study, 48% of youth randomized to TPM completed the 6-week trial (n = 19), compared with 77% of youth who received placebo (n = 20). Adverse medication side effects were the most common reason for withdrawal among participants in the TPM group. Topiramate was superior to placebo in reducing the number of grams of cannabis smoked per use day, but did not improve abstinence rates.^14^ To our knowledge, the efficacy of TPM for the treatment of co-occurring cannabis use and bipolar disorders has not been systematically assessed.

With these considerations in mind, we conducted a 16-week, double-blind, randomized, placebo-controlled investigation of adjunctive TPM for the treatment of cannabis use and manic symptoms in adolescents with co-occurring bipolar and cannabis use disorders.

## Method

### Study Participants

A total of 90 adolescents and young adults, 12 to 21 years of age, presenting with a manic or mixed episode associated with bipolar I disorder and a co-occurring cannabis use disorder (abuse or dependence), were recruited from inpatient and outpatient settings, to participate in this 16-week randomized, double-blind, placebo-controlled, parallel-design investigation evaluating the efficacy and tolerability of TPM vs placebo (adjunctive to quetiapine and therapy) for the treatment of cannabis use and mania associated with bipolar disorder. We conducted a 16-week trial to provide adequate time to titrate TPM to a therapeutic dose and to evaluate its efficacy. Subjects participated in baseline diagnostic assessments using the *DSM-IV-TR*–based Washington University in St. Louis Kiddie Schedule for Affective Disorders and Schizophrenia,^15^ and the *DSM-IV-TR*–based Semi-Structured Assessment for Genetics of Alcoholism—Version II,^16^ performed by a trained clinician with demonstrated diagnostic interrater reliability (κ >0.9). During the diagnostic assessment, we evaluated the temporal relationship between the onset of bipolar I disorder (defined as the onset of the first mood episode) and cannabis use/cannabis use disorder. We also assessed the temporal relationship between the occurrence of mood symptoms and cannabis use. We made a diagnosis of an independent bipolar I disorder when the preponderance of information suggested the following: (1) the onset of mood symptoms occurred distinctly separate from cannabis use; and (2) the pattern of mood symptoms occurred independent of cannabis use. Because each study participant met criteria for an independent bipolar I disorder, their current manic episode was determined to be related to bipolar I disorder rather than to cannabis use. All participants were required to have an intelligence quotient (IQ) of ≥70 as assessed by the Wechsler Abbreviated Scale of Intelligence.^17^ Participants were also required to have a Young Mania Rating Scale (YMRS)^18^ baseline score of ≥16, a diagnosis of cannabis abuse or dependence, and a minimum rate of cannabis use of twice per week on average during the 28 days prior to screening.

Participants were excluded if they met the following criteria: (1) were acutely intoxicated at screening or baseline; (2) had manic or depressive symptoms resulting from intoxication or withdrawal from drugs or alcohol; (3) had an unstable medical or neurological disorder; (4) had a positive pregnancy test result or were lactating; (5) had a history of nephrolithiasis; (6) had a known history or family history of glaucoma; (7) were court ordered to treatment; (8) had significant suicidal ideation as defined by a score of >3 on the Children’s Depression Rating Scale—Revised^19^ suicide item, or any serious suicide attempt within the prior 60 days as judged by the investigator; or (9) had a history of non-response or poor tolerability to TPM or quetiapine.

This study and all procedures were approved by the institutional review boards at the Cincinnati Children’s Hospital Medical Center and the University of Cincinnati and were conducted in accordance with the International Conference on Harmonization Good Clinical Practice. Before any study procedure, all adult subjects provided written informed consent, whereas all minors provided written informed assent and their legal guardians provided written informed consent, respectively. This trial was registered on clinicaltrials.gov (NCT00393978).

### Study Design

After completing the screening and baseline visit assessments, subjects meeting study criteria were randomized either to the TPM group or to the placebo group. Randomization assignment was stratified by type of cannabis use disorder (abuse vs dependence) and sex. At baseline, TPM was initiated at a dose of 25 mg twice daily and was increased to 75 mg twice daily by day 21 to a maximum daily dose of 150 mg twice daily. Topiramate was flexibly dosed; however, a minimum dose of 75 mg twice daily, reached by day 21, was required. Quetiapine was simultaneously initiated at a dose of 100 mg and increased daily by 100 mg to a target daily dose of 400 mg (maximum of 800 mg daily). The quetiapine dose was not increased by more than 100 mg per day. Beginning at week 1, subjects participated in a single Motivational Interview and 16 sessions of Compliance Enhancement Therapy as a minimal standard psychosocial intervention for substance use disorders.

During the 16-week study period, participants had weekly study visits to assess symptom response and for the presence of adverse side effects using the Side Effects Form for Children and Adolescents (SEFCA).^20^ Pre-treatment and post-randomization assessments included the following: Timeline Followback (TLFB)^21^; YMRS^18^; Children’s Depression Rating Scale—Revised^19^; and Marijuana Craving Questionnaire (MCQ).^22^ The MCQ is a 12-item self-report scale that evaluates explanations for marijuana craving. The subscales include the following: (1) compulsivity (ie, the inability to control marijuana use); (2) emotionality (ie, the use of marijuana in anticipation of relief from a negative mood or withdrawal); (3) expectancy (ie, the anticipation of positive outcomes from smoking marijuana); and (4) purposefulness (ie, planning and intending to use marijuana to attain a positive outcome).^23^ The screening assessment also included a complete medical history, physical examination, laboratory tests, vital signs (blood pressure, pulse, weight, height, and temperature), serum pregnancy test, fasting glucose, fasting lipid profile, renal profile, complete blood count, liver function tests, thyroid-stimulating hormone, and 12-lead electrocardiography. Vital signs were recorded at every study visit and at week 16 (or at termination). An 8-panel urine toxicology screen was performed at screening, baseline, and at the end of weeks 2, 4, 6, 8, 10, 12, 14, and 16 (or at termination).

### Statistical Analyses

Our primary outcome measure was change in weekly cannabis use, measured as change in joint equivalents using the TLFB, over the 16-week treatment period. Our secondary outcome measure was the baseline-to-endpoint total score change in the YMRS. For weekly cannabis use, a generalized linear mixed model was used that assumed the counts to follow a Poisson distribution with a logarithmic link function. The arithmetic average of the weekly marijuana use for the 28-day baseline period was used as a covariate, and the use in each week of the 16-week follow-up period were the repeated dependent measures. Unstructured means (ie, analysis of variance [ANOVA]) modeling was used, as well as a mixed regression model for linear change in each group over time on treatment. A similar approach was used for the number of use days per week. Furthermore, each model was run on the observed data (including partial series for subjects who did not complete) as well as on a last observation carried forward (LOCF) data set. Comparisons were performed on differences of baseline-adjusted weekly logarithmic mean use between groups (for the ANOVA model) and on the differences of on-treatment slopes between groups (for the regression model). Results were back-transformed to the original scale for ease of interpretations so that average values given are geometric means (ie, the arithmetic average of logged values, exponentiated). All analyses were conducted using SAS software, version 9.4.

## Results

### Participants and Disposition

**Figure 1:**
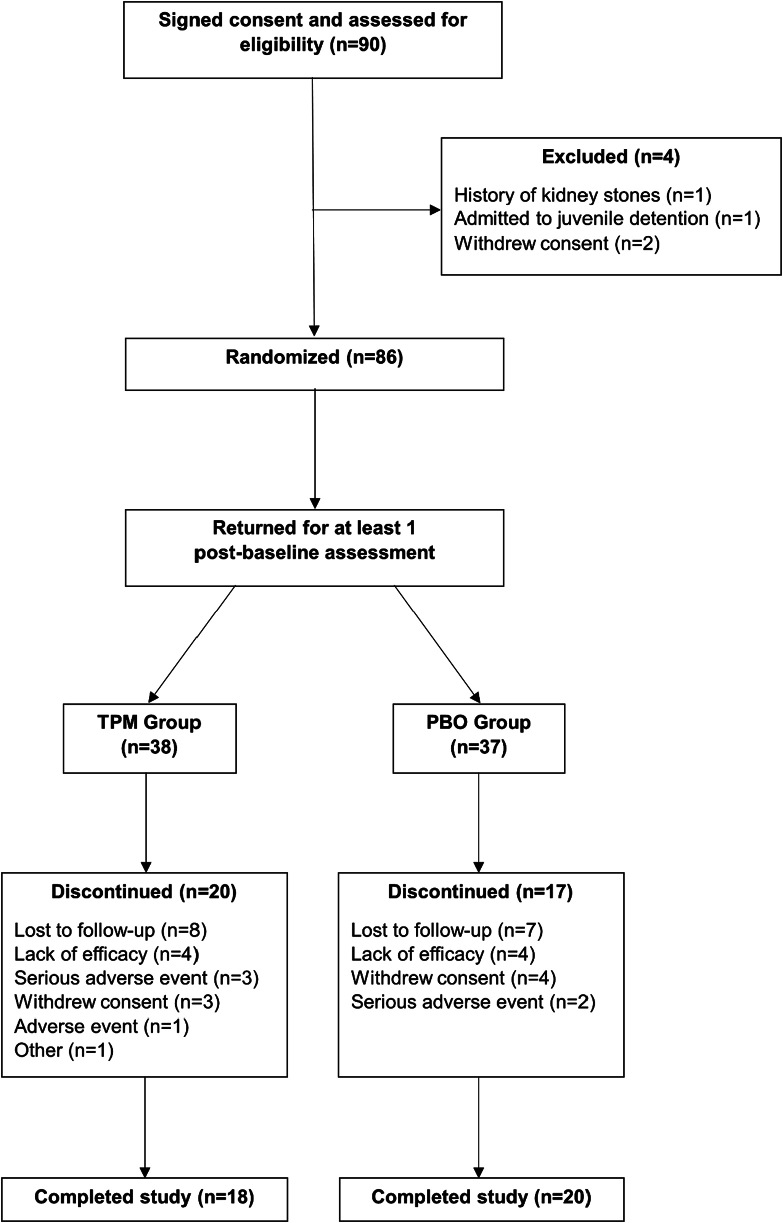
CONSORT Diagram of Study Participant Flow ***Note:****PBO = placebo; TPM = topiramate.*

A total of 90 participants were enrolled (Figure 1). Four participants did not meet study criteria, and 11 did not have a post-baseline visit. Therefore, 75 participants (TPM, n = 38; placebo, n = 37) were included in our intent-to-treat sample. The overall completion rate for the 16 weeks of randomized treatment was 51%. The groups did not differ significantly in completion rates (TPM n = 18, 47% and placebo n = 20, 54%, *p* = .97) or in time to any-cause discontinuation (*p* = .21) based on comparison of Kaplan–Meier survival curves by the log-rank test. Mean duration of follow-up in weeks was 10.89 for the TPM group and 12.84 for the placebo group. Baseline demographic and clinical characteristics were similar between treatment groups (Table 1)

**Table 1:** Baseline Clinical and Demographic Variables by Treatment Group

	TPM (n = 38)	Placebo (n = 37)	*p*^a^
Age, y, mean (SD)	17.7 (2.1)	17.1 (2.1)	.21
Sex, female, n (%)	20 (53)	18 (49)	.82
Race, White, n (%)	31 (82)	33 (89)	.52
Hospitalization, current, n (%)	15 (39)	20 (54)	.25
Mixed (vs manic) episode, n (%)	34 (89)	32 (86)	.74
Age onset of bipolar disorder, y, mean (SD)	13.9 (2.3)	13.9 (2.6)	.97
Psychosis, n (%)	10 (26)	7 (19)	.58
Attention-deficit/hyperactivity disorder, n (%)	21 (55)	19 (51)	.82
Stimulant use, concomitant, n (%)	5 (13)	47 (19)	.54
Anxiety disorder, n (%)	16 (42)	15 (41)	.99
Conduct disorder, n (%)	19 (50)	17 (46)	.82
Cannabis abuse, n (%)	12 (32)	10 (27)	.80
Cannabis dependence, n (%)	26 (68)	27 (73)	.80
Age onset of cannabis abuse or dependence, y	14.9 (2.0)	14.4 (1.8)	.23
Alcohol abuse or dependence, n (%)	20 (53)	25 (68)	.24
Nicotine dependence, n (%)	24 (63)	24 (65)	.99
Young Mania Rating Scale score, mean (SD)	24.5 (4.9)	24.1 (6.1)	.75
Children’s Depression Rating Scale–Revised score, mean (SD)	37.1 (11.1)	36.1 (7.6)	.67
Weight, lb, mean (SD)	147.4 (42.2)	165.2 (52.4)	.11
Body mass index, kg/m^2^, mean (SD)	23.7 (5.6)	26.4 (8.0)	.10
Marijuana Craving Questionnaire subscales, mean (SD)			
Compulsivity	2.9 (1.4)	3.0 (1.4)	.74
Emotionality	5.8 (1.4)	6.1 (1.1)	.31
Expectancy	4.8 (1.2)	5.4 (1.7)	.06
Purposefulness	4.7 (1.9)	4.8 (1.9)	.91

Note: TPM = topiramate.

a Test statistic was Fisher exact test for categorical variables and Student *t* test for continuous variables.

### Study Medications

The median daily quetiapine dose was 426 mg (SD, ±142) in the TPM group and 431 mg (SD, ±125) in the placebo group (*p* = .916). The median daily TPM (or placebo) “dose” was 208 mg (SD, ±87) in the TPM group and 278 mg (SD, ±57) in the placebo group (*p =* .005).

### Efficacy

#### Cannabis Use

**Figure 2:**
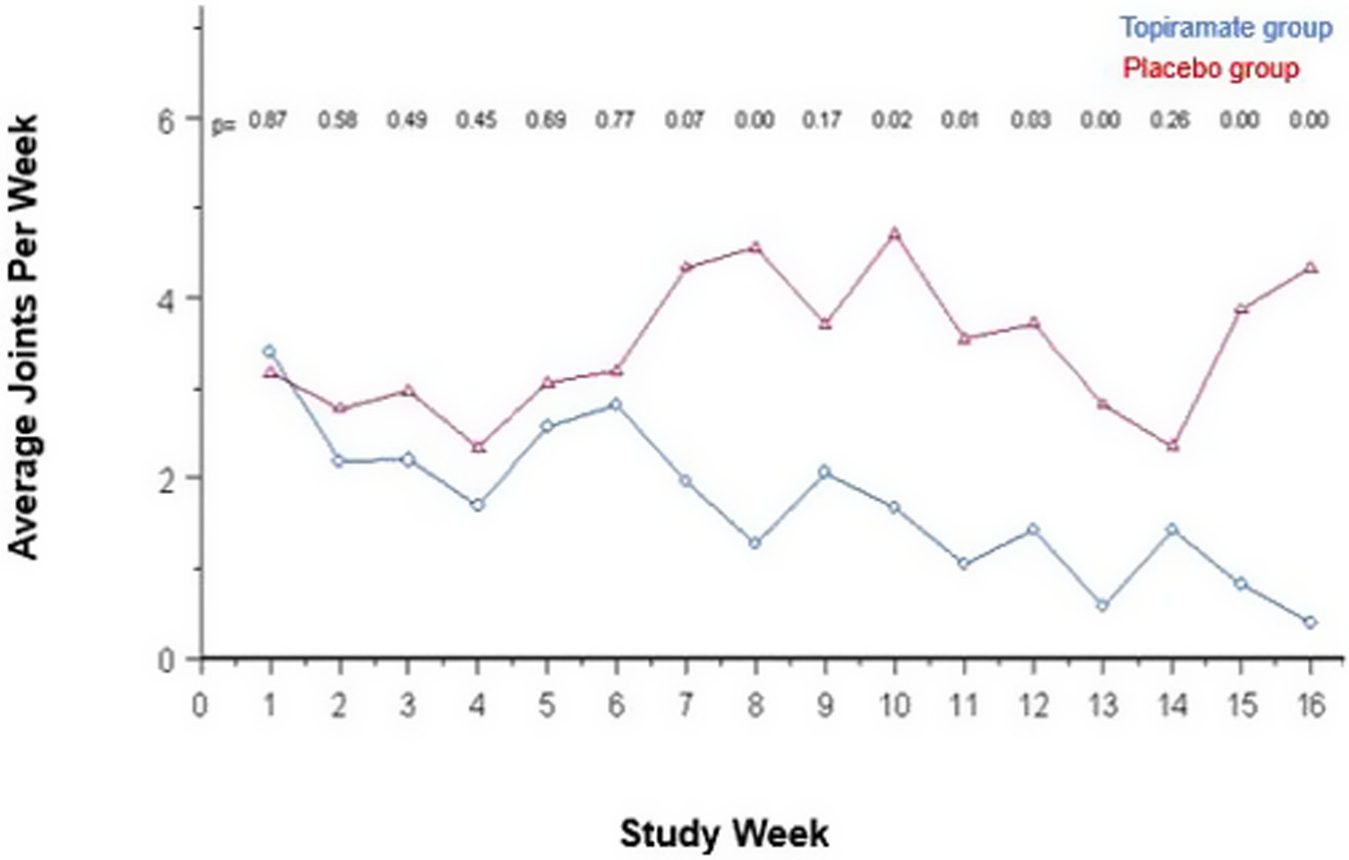
Baseline-Adjusted Cannabis Use Per Week by Treatment Group ***Note:****Treatment by week interaction effect (*p *< .001)*.

The mean absolute baseline weekly cannabis use was 17.0 (SD, ±17.1) joints per week in the TPM group and 22.0 (SD, ±21.1) in the placebo group (*p* = 0.26), whereas the estimated geometric mean weekly use was 11.1 joints per week in the TPM group and 14.4 joints per week in the placebo group (2-sample *t* test: *p* = .27). Cannabis use decreased rapidly in both groups during the first week of treatment, and there was a significant time-by-treatment effect over the 16-week treatment period, with baseline-adjusted treatment differences in favor of the TPM group over time (ie, by generalized Poisson ANOVA model likelihood ratio test; *p* < .001) (Figure 2). By week 8, the TPM group was using fewer joints per week (*p* = .003), and this difference remained present at week 16 (*p* < .001) (Figure 2). The presence of comorbid attention-deficit/hyperactivity disorder (ADHD) predicted a greater reduction in joints used per week in the TPM group (*R*^*2*^ = 0.125; *F* test; *p* = .029).

At week 16, the estimated average cannabis use was 0.4 joints per week in the TPM group (a 96% decrease in use) vs 4.3 joints per week in the placebo group (a 70% decrease in use). Correspondingly, at week 16, the LOCF data demonstrated a similar pattern of decrease in cannabis use: 0.9 joints per week in the TPM group vs 3.6 joints per week in the placebo group (2-sample *t* test; *p* = .003) with decreases in use of 92% vs 75%, respectively (effect size for percent change; *d* = 0.39). This finding was further supported by the regression analysis, demonstrating a significantly more negative slope (decreased rate of use) in the TPM group compared with the placebo group (*F* test; *p* < .001).

**Figure 3:**
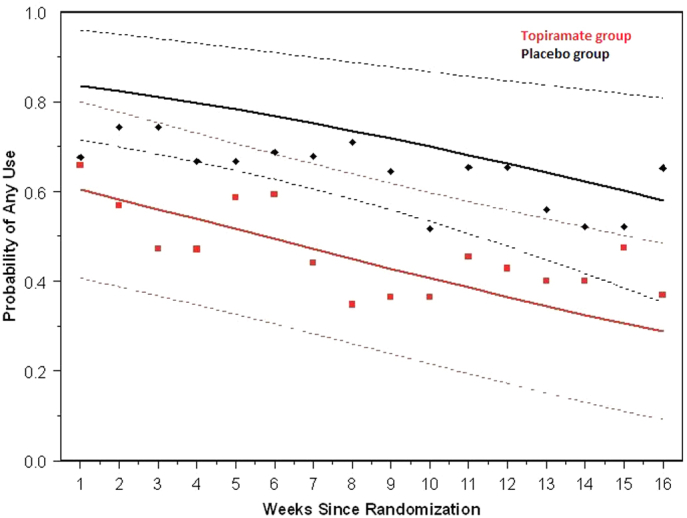
Weekly Probability of Cannabis Use Over Time ***Note:****The topiramate group demonstrated a lower probability of use (*p *= .03).*

At baseline, there was no significant difference between groups in the percentage of cannabis use days (58%, or about 4 use days per week, in both groups; *p* = .95). Both groups displayed a rapid decrease in use days in the first week of treatment. Although there was no significant interaction over time (*p* =.14), there was a lower rate of use days in the TPM group throughout the treatment period (*p <* .006). On average, subjects in the TPM group used cannabis on 14.5% of treatment days, or about 1 day per week, whereas those in the placebo group used on 28.1% of treatment days, or about 2 days per week. In addition, although both treatment groups decreased their weekly probability of any cannabis use significantly over time (*p* = .0002), the TPM group demonstrated a lower probability of use (*p* = .03) (Figure 3). Finally, there was no difference between the TPM group (71%) and the placebo group (81%) in the average proportion of urine drug screens that tested positive during the study (*p* = .15). There was a trend for a lower rate of a positive urine drug screen in the TPM group (61%) compared with the placebo group (81%) at the week 16 or early-termination visit (*p* = .07).

On the MCQ, there were no differences between groups in changes in compulsivity (*p* = .16), emotionality (*p* = .91), or expectancy (*p* = .25) from baseline to endpoint. However, there was a significant change in purposefulness (*p* = .03), indicating that the TPM group demonstrated a reduction in cannabis use toward intentional attainment of a positive outcome (Table 2).

**Table 2:** Comparison of Change in Clinical Variables Between Topiramate and Placebo Groups

	TPM (n = 38)	Placebo (n = 37)	*p*^a^
Change in Young Mania Rating Scale score, mean (SD)	13.5 (9.0)	15.5 (9.0)	.342
Change in Children’s Depression Rating Scale–Revised score, mean (SD)	10.6 (13.1)	9.6 (10.5)	.699
Change in weight, lb, mean (SD)	0.4 (8.7)	5.5 (8.1)	.010
Change in body mass index, kg/m^2^ mean (SD)	0.1 (1.4)	0.9 (1.2)	.008
Marijuana Craving Questionnaire, change in subscale score, mean (SD)			
Compulsivity	−0.7 (1.7)	−0.2 (1.2)	.160
Emotionality	−0.4 (1.8)	−0.4 (1.3)	.910
Expectancy	−1.3 (1.8)	−0.8 (1.6)	.250
Purposefulness	−1.7 (2.5)	−0.6 (1.9)	.030

Note: TPM = topiramate.

a Test statistic was Student *t* test.

#### Mood Symptoms and Severity

There was no difference in mean baseline YMRS (*p* = .75) total scores between groups (Table 1). Total YMRS scores within each group declined significantly over time (*p* < .0001). There was no significant difference between groups in change in YMRS total score (*p* = .34) (Table 2), response rates (≥50% reduction in the YMRS score; *p* = .27), or remission rates (YMRS score <12; *p* = .88) (Table 2). Although there was not a statistically significant group difference in manic symptom reduction over time, there was a trend for a more rapid decrease in the TPM group.

We conducted a preliminary time-lagged analysis to examine the relationship between change in mood symptoms and change in cannabis use. Change in cannabis use at time t-1 was significantly predictive of change in mood symptoms at time t (*p* < .001 for YMRS score and *p* < .001 for CDRS-R score). In contrast, change in mood ratings at time t-1 was not as predictive of change in cannabis use at time t (*p* = .06 for the YMRS and *p* = .04 for the CDRS-R), suggesting that reducing cannabis use is important for improving mood symptoms.

#### Alcohol and Nicotine Use

At baseline, there were no differences between groups in the percentage of subjects who met criteria for alcohol abuse/dependence (*p* = .24) or nicotine dependence (*p* = .99)

(Table 1). There was a significant treatment-by-time effect for the amount of alcohol used (*p* < .001) such that participants in the TPM group drank less alcohol by week 16 (*p =* .006) than those in the placebo group. In addition, there was a main treatment effect for nicotine use (*p* = .033); the TPM group smoked fewer cigarettes on average over the treatment period compared with the placebo group.

### Safety and Tolerability

On the SEFCA, more participants in the TPM group experienced appetite decrease (63% vs 87%, *p* = .03) and excitement *(*13% vs 0%, *p* = .03) compared with the placebo group. There were 3 serious adverse events observed in the TPM group, namely self-reported suicidal ideation (n = 3), and 2 serious adverse events in the placebo group, namely pregnancy (n =1) and suicide attempt (n = 1). During the course of study participation, there was no difference in self-reported suicidal ideation (*p* = .90) between groups.

### Weight and Body Mass Index

There was not a significant group difference in baseline weight *(p* = .11) or body mass index (BMI) (*p* = .10) (Table 1). Increase in weight from baseline to endpoint was greater in the placebo group compared with the TPM group (*p* = .01) (Table 2). Moreover, there was a significant treatment-by-time effect (*p* = .0005); the increase in BMI over time was greater in the placebo group compared with the TPM group (*p* = .008) (Table 2).

### Laboratory Values

The TPM group demonstrated a non–clinically meaningful, but significantly, greater increase in chloride (mean change [SD] = 2.65 [3.33] mEq/L) from baseline to endpoint than the placebo group (−0.20 [2.69] mEq/L*; p* = .002). There were no other group differences in change in laboratory values over the treatment period, including in glucose (*p* = .53), insulin (*p* = .64), high-density lipoprotein (*p* = .12), or low-density lipoprotein (*p* = .42).

## Discussion

To our knowledge, this is the first prospective, placebo-controlled study evaluating the treatment of adolescents with co-occurring bipolar I disorder and cannabis use disorder.

Treatment with TPM adjunctive to quetiapine and a Motivational Interview and Compliance Enhancement Therapy was associated with improvement in manic symptoms, as well as a greater decrease in cannabis use and less weight gain compared with placebo adjunctive to quetiapine and a Motivational Interview and Compliance Enhancement Therapy.

In contrast to extant studies of youth without bipolar disorder in which TPM reduced the quantity, but not frequency, of cannabis use,^14^^,^^24^ treatment with TPM decreased both the quantity and frequency of use. Craving, a core feature of cannabis use disorder,^25^ is critical in the development and maintenance of cannabis misuse ^26^ and predicts the quantity of next-moment cannabis use in adolescents.^24^ Topiramate is postulated to reduce craving via agonism of GABA and antagonism of non-NMDA glutamate to decrease mesocorticolimbic dopamine release.^12^ In contrast to prior findings suggesting that TPM does not have an impact on craving in adolescents without bipolar disorder,^24^ we found that treatment with TPM was associated with a decrease in purposefulness, a core craving-related factor that corresponds to planned cannabis use to generate a positive outcome; indeed, prior studies have postulated that purposefulness predicts cannabis use in young adults.^27^ Accordingly, our findings suggest that the benefit of TPM for cannabis use disorder may be mediated by reduced craving.

Interestingly, in the TPM group, we found a positive association between comorbid ADHD and a greater reduction in number of joints smoked. This was unexpected, given the strong link between ADHD and cannabis use disorder.^28^ Both ADHD and cannabis use disorder involve abnormal reward processing,^29^ and ADHD may be associated with underactivity of the dopamine reward pathway.^30^ Cannabis increases dopamine release via GABAergic disinhibition of dopamine neurons,^29^^,^^30^ conferring a potential reduction in ADHD symptoms and supporting a “self-medication” explanation to the link between the 2 disorders.^31^ TPM is associated with cognitive complaints^32^ and may modulate the reinforcing effects of cannabis by reducing corticomesolimbic dopamine release.^12^ Therefore, 1 possibility is that it could worsen ADHD symptoms, leading to increased cannabis use. Alternatively, as cannabis use in adolescents with bipolar disorder is associated with working memory deficits,^33^ and as individuals with (vs without) ADHD are well known to experience deficits in working memory,^34^ it is possible that by reducing cannabis use, TPM reduced ADHD symptoms in the present study and attenuated the “self-medication” benefit of cannabis, leading to a decrease in joints used.

In the present study, treatment with adjunctive TPM did not confer greater improvement in manic and/or depressive symptoms compared with placebo. Prior studies suggest that TPM may be effective for treating mania in youth.^35^, ^36^, ^37^, ^38^ However, consistent with other studies evaluating treatment with adjunctive TPM in adolescents with bipolar disorder and without comorbid cannabis use disorder,^32^^,^^34^ we did not find a greater reduction in manic or depressive symptoms when TPM was added to a second-generation antipsychotic (SGA), although this may have been due to a threshold effect. Our findings suggest a bidirectional relationship between cannabis use and mood. However, our results indicate that decreasing cannabis use predicts mood improvement more strongly than reducing mood symptoms affects cannabis use.

There is a dearth of evidence regarding pharmacologic intervention for alcohol and tobacco use in adolescents with bipolar disorder, and little is known about the impact of TPM. Our results suggest that treatment with TPM in adolescents with bipolar disorder is associated with less alcohol and cigarette use. Studies in adults without bipolar disorder have demonstrated the mostly positive impact of TPM on alcohol craving^39^, ^40^, ^41^ and consumption.^39^, ^40^, ^41^, ^42^, ^43^ In adults with bipolar disorder, a case report demonstrated an appreciable reduction in alcohol drinking associated with adjunctive TPM^44^; however, in a controlled study, TPM did not reduce the quantity or frequency of alcohol use.^45^ Similarly, although there is weak evidence for benefit of TPM for smoking cessation in adults without bipolar disorder^2^^,^^46^^,^^47^ and with schizoaffective disorder, bipolar type,^48^ the impact of TPM on cigarette use has not been evaluated in adolescents with or without bipolar disorder. To our knowledge, this study is the first to investigate the impact of TPM on alcohol and cigarette use in adolescents with bipolar and cannabis use disorder, and our findings suggest that TPM is a promising intervention for reducing the use of both substances.

Roughly half of our participants met criteria for conduct disorder at study baseline (Table 1). Although bipolar and conduct disorder are independent risk factors for the development of substance use disorders in adolescents, among adolescents with bipolar disorder, those with substance use disorders demonstrate a higher number of conduct symptoms compared with those without a substance use disorder (*p* = .04). Our findings are consistent with the high prevalence of conduct disorder observed among adolescents with bipolar disorder (ie, 55% vs 8% without bipolar disorder; *p* < .001)^2^ and offer further evidence of the high prevalence of conduct disorder in adolescents with bipolar disorder and comorbid cannabis use disorder.

Although its exact mechanism of action is not known, TPM may mitigate SGA-related weight gain by reducing food craving and improving impulse control.^32^ We found that TPM was associated with less weight gain and lower BMI over the treatment period. Our findings are consistent with adult open-label^49^ and double-blind^50^, ^51^, ^52^ studies as well as meta-analyses^53^, ^54^, ^55^ that suggest that TPM is effective for reducing and preventing SGA-associated weight gain. Our findings are also consistent with investigations of adjunctive TPM in SGA-treated youth with bipolar disorder demonstrating a positive effect on anthropometric measures,^32^^,^^37^^,^^38^^,^^56^ and suggest that adjunctive TPM remains beneficial in the presence of comorbid cannabis use disorder. Consistent with prior studies,^32^^,^^37^ we did not find group differences in high-density lipoprotein, low-density lipoprotein, insulin, and/or glucose values associated with TPM in this 16-week study. Longer studies may be necessary to observe the benefit of TPM on these measures.

Commonly reported side effects of TPM include reduced appetite, renal calculi, decreased cognition, and slowed and dulled thinking.^32^ Although not unexpected or clinically meaningful, there was a greater increase in chloride in the TPM group, likely due to the inhibitory effect of TPM on carbonic anhydrase. In the present study, appetite and excitement occurred more frequently in the TPM group, and, consistent with other studies of adolescents with bipolar disorder (without cannabis use disorder), ^13^^,^^32^^,^^35^^,^^36^^,^^38^ treatment with TPM was safe and well tolerated.

In contrast to the prior placebo-controlled study of TPM treatment for adolescents (without bipolar disorder) who used cannabis, in which adverse events disproportionately contributed to attrition,^14^ we did not observe a significant between-group difference in the attrition rate. The high overall attrition rate (49%) in our 16-week study is commensurate with comorbid adolescent studies and suggests a direct relationship between study length and attrition. In a shorter, 6-week investigation of lithium for treatment of adolescents with bipolar disorder and substance dependence, the attrition rate was low (16%),^9^ whereas in a 12-month study evaluating family-focused therapy for adolescents with bipolar and substance use disorders, the attrition rate was 80%.^11^ In addition, in an investigation of pharmacologic intervention for adults and older adolescents with comorbid bipolar and substance use disorders, the attrition was high in the initial short term (79% over 6 weeks) and subsequent long-term (74% of the remaining participants over 6 months) phases of the study.^10^ As in our study, discontinuation was commonly due to treatment non-response or loss to follow-up, suggesting that significant psychosocial stress and illness burden may also account for high attrition in this comorbid population. Clinically, this suggests that adherence to a course of treatment may decrease with time, and/or that greater illness burden and psychosocial stress in a comorbid population may present greater challenges to treatment adherence.

There are several limitations to consider when interpreting our results. First, we evaluated a predominantly White study population, which may limit the generalizability of our results. In light of known ethnic and racial disparities in treatment access for minoritized groups with comorbid substance use and mental health disorders,^57^ our recruitment from a clinical population may have limited the diversity of our sample. Future studies using broader recruitment methods may serve to enhance diversity in a comorbid study population. Second, we did not formally assess cognition or follow ADHD symptom changes over time. Future studies of TPM in this population should include formal neurocognitive assessments and ADHD symptom ratings and should examine the temporal relationship among cannabis use, cognitive changes, and ADHD and mood symptom changes. Finally, given the relatively short duration of our investigation, we cannot draw conclusions about the long-term impact of TPM on cannabis use in adolescents and young adults with bipolar disorder.

In conclusion, we found TPM to be a safe and well-tolerated intervention that can reduce the quantity and frequency of cannabis use in adolescents with bipolar disorder. Future long-term studies to corroborate our findings, as well as to evaluate the efficacy of TPM for other co-occurring substance use disorders in youth with bipolar disorders, are needed.

## CRediT authorship contribution statement

**Jenni E. Farrow:** Writing – review & editing, Writing – original draft, Formal analysis, Data curation. **Melissa P. DelBello:** Writing – review & editing, Writing – original draft, Visualization, Validation, Supervision, Resources, Project administration, Methodology, Investigation, Funding acquisition, Formal analysis, Data curation, Conceptualization. **Luis R. Patino:** Writing – review & editing, Supervision, Project administration, Methodology, Data curation. **Thomas J. Blom:** Writing – review & editing, Methodology, Formal analysis, Data curation. **Jeffrey A. Welge:** Writing – original draft, Methodology, Funding acquisition, Formal analysis, Data curation.
